# Knowledge, attitudes and practices on adolescent vaccination among parents, teachers and adolescents in Africa: a systematic review protocol

**DOI:** 10.1186/2046-4053-3-100

**Published:** 2014-09-09

**Authors:** Leila H Abdullahi, Benjamin M Kagina, Tali Cassidy, Esther F Adebayo, Charles S Wiysonge, Gregory D Hussey

**Affiliations:** 1 Vaccines for Africa Initiative, Institute of Infectious Disease and Molecular Medicine, University of Cape Town, Cape Town 7925, South Africa; 2 Division of Medical Microbiology, Department of Clinical Laboratory Sciences, University of Cape Town, Cape Town 7925, South Africa; 3 Centre for Evidence-based Health Care and Division of Community Health, Faculty of Medicine and Health Sciences, Stellenbosch University, Cape Town 7925, South Africa; 4 School of Public Health and Family Medicine, University of Cape Town, Cape Town 7925, South Africa

**Keywords:** Adolescents, Parents, Teachers, Knowledge attitudes and practice, Vaccination barriers, Africa

## Abstract

**Background:**

Vaccines are the most successful and cost-effective public health interventions available to avert vaccine-preventable diseases and deaths. Despite progress in the field of adolescent health, many young people in Africa still get sick and die from vaccine-preventable diseases due to lack of vaccination. Parents, adolescents and teachers are key players with regard to implementation of adolescent vaccination policies. Therefore, understanding their knowledge, attitudes and practices towards adolescent vaccination may provide clues on what can be done to improve vaccine uptake among adolescents. The aim of this study is to conduct a qualitative and quantitative systematic review on knowledge, attitudes and practices on adolescent vaccination among parents, teachers and adolescents in Africa.

**Methods:**

We will include both quantitative and qualitative primary studies. Eligible quantitative studies include both intervention and observational studies. Qualitative studies to be included are focus group discussions, direct observations, in-depth interviews and case ethnographic studies. We will search PubMed, Cochrane Central Register of Controlled Trials, Scopus, Web of Science, WHOLIS, Africa Wide and CINAHL for eligible studies with no time and language limits. We will also check reference lists of included studies for other eligible reports. Two authors will independently screen the search output, select studies and extract data, resolving discrepancies by consensus and discussion. We will analyse qualitative data using thematic analysis where applicable, and quantitative studies findings will be presented in a narrative synthesis form based on the outcomes.

**Discussion:**

The findings from this systematic review will guide the identification of gaps on knowledge, attitudes and practices among the key role players on adolescent vaccination. We anticipate that our findings will guide the development of adolescent-focused vaccination policy in Africa, which is virtually non-existent at present.

**Systematic review registration:**

This review is registered with PROSPERO, registration number CRD42014010395.

## Background

Routinely, in many African countries, most vaccines recommended by the World Health Organisation (WHO) are administered early in life at a health clinic [1]. Vaccine coverage is thus high in infancy in most countries [1,2]. However, some vaccines induce short-lived immunity that wanes over time leading to a susceptible population later in life [3-5]. In such cases, it is recommended that booster doses of the vaccines previously administered in infancy or childhood should be provided later in life (for example during adolescence) to maintain vaccine-induced immunity [3-6]. It is critical that the coverage of primary and booster vaccine doses is optimal. In general, the childhood immunisation programmes have achieved substantial vaccination coverage. As a result, an estimated 2.5 million child deaths a year are avoided [4]. However, routine adolescent immunisation programmes are non-existent in most African countries [7]. As a consequence, adolescents in these settings have suboptimal immunity against some vaccine-preventable diseases [4].

Tetanus and pertussis are examples of antigens whose vaccine-induced immunity wanes over time, requiring booster vaccines later in life, preferably during adolescence period [3,5]. New vaccines under development like human immunodeficiency virus (HIV) and tuberculosis (TB) vaccines are a key element of the Global Vaccine Action Plan 2011–2020 [6] and may target adolescents as their primary routine population [3,4]. Therefore, studies focussing on improved understanding towards adolescent vaccination are critical.

In Africa, several countries like Rwanda, Uganda, Cameroon, Tanzania, Lesotho and South Africa, have introduced human papillomavirus (HPV) vaccine to prevent cervical cancer through school-based programme [8-15]. The programme aims to vaccinate pre-adolescent and adolescent girls against the HPV [16]. Such programmes could serve as a basis for the development of adolescent vaccination programme [17]. Therefore, studies investigating factors associated with improved uptake of vaccines among adolescents will help to guide the development of adolescent vaccination policies in these settings.

According to WHO, adolescents refer to young persons aged 10 to 19 years [1,18]. The adolescent group may be categorised in three subgroups, i.e. early (10–13 years old), mid (14–16 years old) and late (17–19 years of age) adolescence [1,18]. Interestingly, at the beginning of 2014, global statistics showed that there are 1.8 billion young people aged 10–24 years, representing 25% of the world's population [19]. This suggests that optimal vaccination coverage to this large number of young persons would achieve an enormous public health benefit that includes reduced disease transmission and a healthier population at a later age in life [3,4].

Targeting adolescents with catch-up and booster vaccines is critical for three reasons: achievement of primary immunisation of new vaccines, catch-up on missed vaccinations and boosting of the waning immunity [3,20]. The WHO recommends vaccinating 11 or 12 years old against *Neisseria meningitidis*, *Bordetella pertussis* and HPV [21]. Additionally, current guidelines by WHO recommends vaccination against measles, mumps, rubella, varicella, hepatitis B and polio for those who have not previously received these vaccines as a catch-up [5,21,22]. Despite these recommendations, in Africa, adolescent vaccination coverage remains largely suboptimal.

Vaccinating adolescents has several challenges [23,24]. The challenges include lack of knowledge about vaccines and vaccine-preventable diseases among key role players like parents and teachers [20,22,23,25-30]. Parents (one who nurtures and raises a child or a relative who plays the role of guardian) [31] are routinely involved in the decision-making process of vaccine administration to their children. Therefore, parental involvement, including knowledge and attitude towards vaccination can influence the willingness of adolescents to be vaccinated [25,26,28,29]. While adolescents, and in particular early adolescents (10–13 years old) [1,18], may not have an independent final decision getting vaccinated, educating this population about vaccination may have long-term implications on the vaccine uptake rates among this age group [30,32]. Educating adolescents can positively influence their knowledge and attitudes towards vaccination and may have several long-term benefits.

First, as future parents, educated adolescents are more likely to encourage their children to be vaccinated [32]. Second, as future adults, the adolescents are more likely to be acquiescent to vaccinations [32]. Third, educated adolescents may be able to better inform and influence parents and peers on vaccinations than the uneducated peers [32]. Finally, teachers (professional person who teaches or instructs) [33] may play a crucial role in adolescent vaccination uptake since the school-based vaccination programmes where they may exist may be a popular platform to vaccinate adolescents [32]. Hence, adequate knowledge and positive attitudes towards vaccination among parents, teachers and adolescents may improve uptake of vaccines among adolescents [23,25-29].

Vaccination knowledge refers to the understanding of any related topic on adolescent vaccines [34]. Attitude towards vaccination refers to the feelings towards adolescent vaccines, as well as any preconceived ideas that one may have towards vaccination [34]. In our study, practice refers to the ways in which one demonstrates the knowledge and attitude (and any other influences) through actions [34].

Recently, a number of studies on the knowledge and attitude among parents, adolescents and healthcare workers towards HPV vaccines have been published [23,25-29]. Knowledge, attitudes and practices (KAP) towards HPV vaccine is, however, different from KAP towards other adolescent vaccines. For example, HPV may be viewed as dreadful disease thus increasing acceptance of HPV vaccines [26-28]. This may not be the case with other vaccines such as DPT. We are not aware of published systematic reviews on the knowledge, attitudes and practice of parents, teachers and adolescents on all adolescent immunisation, especially in Africa. We will therefore conduct a systematic review on this topic.

### Objectives

(a). The primary objective of this study is to assess the KAP on adolescent vaccination among parents, teachers and adolescents in Africa.

(b). The secondary objective of this study is to assess the effect of KAP on adolescent vaccine uptake in Africa.

## Methods

This review protocol has been published in the PROSPERO International Prospective Register of systematic reviews (http://www.crd.york.ac.uk/PROSPERO), registration number CRD42014010395 [35].

## Study eligibility criteria

### Study design

Quantitative studies to be included are randomised control trials (RCTs), controlled before-and-after studies (CBAs), interrupted time series designs (ITS), cohort studies, case–control studies, and cross-sectional studies. Qualitative studies are defined as those that use established qualitative methods of data collection such as focus group discussions, in-depth interviews, direct observation, case studies and ethnography and action research.

### Study participants

The study participants are parents, teachers and adolescents.

A parent is defined as one who nurtures and raises a child or a relative who plays the role of guardian. An adolescent is defined as young person aged 10 to 19 years. A teacher refers to a professional person who teaches or instructs.

### Type of intervention

• KAP for interventional studies.

• No intervention applicable for all the other eligible studies in this review.

### Type of outcome measures

#### Primary outcomes

The primary outcomes of this study are knowledge, attitudes and practices of adolescent vaccination among teachers, adolescents and parents.

To measure the outcomes of knowledge, attitudes and practices, the study will collect data on at least one of the following three characteristics: knowledge, attitudes or practices of the target population related to adolescent vaccination, defined as:

• Knowledge: information possessed by parents, adolescents and teachers about adolescent vaccines, evaluated by asking questions about scientific evidence or other issues related to adolescent vaccines and their indications.

• Attitudes: a posture or opinion about adolescent vaccines or vaccination that involves a vaccine-related act or its omission.

• Practices: observable actions of parents, teachers and adolescents in response to adolescent vaccination.

#### Secondary outcome

The secondary outcome of this study is the vaccination coverage (i.e. proportion of adolescents who have received the recommended doses of the vaccine in a study).

### Study settings

We will include studies conducted in any country on the African continent.

### Search strategy

Comprehensive search strategies will be developed for identifying studies examining knowledge, attitudes and practices towards adolescent vaccination among parents, teachers and adolescents in Africa. We will search both published and unpublished articles with no language restrictions from 1950 to 31 August 2014. The peer-reviewed articles in the following electronic databases will be screened: PubMed, Cochrane Central Register of Controlled Trials, Scopus, Web of Science, World Health Organisation Library Information System WHOLIS, Africa Wide and CINAHL. We will search websites and databases for grey materials (Additional file 1: Appendices). The search strategies for electronic databases will incorporate both medical subject headings (MeSH) and free-text terms and will be adapted to suit each individual database using applicable controlled vocabulary (Additional file 1: Appendices). Reference lists of relevant reviews and all eligible papers will also be searched for relevant studies.

### Study selection

Two authors will screen titles and abstracts to select potential eligible studies. Then, the full text of potentially eligible studies will be obtained, and the final selection for inclusion into the review will be conducted by two independent authors. Any disagreements regarding inclusion of studies will be resolved by discussion or by consulting a third author. A PRISMA flow chart will be used to summarise the search and selection of studies for the review. A table of all included studies will be included in the final review, and the reasons for exclusion of studies will also be documented.

### Data extraction

Data will be extracted from selected studies independently by two authors using standardised data extraction forms (Additional file 1: Appendices). Disagreements on study selection and data extraction will be resolved by consensus between the two review authors, failing which a third author will arbitrate. Prior to use, the extraction form will be piloted on at least four studies identified randomly from the list of included studies.

The data extraction will include the following eligibility criteria:

1. Setting of the study (city and country).

2. Study design—RCTs, CBAs, ITS, cohort studies, case–control studies, cross-sectional studies, focus group discussions, in-depth interviews, direct observation, case studies and ethnography and action research.

3. Type of participants—adolescents, caregivers and teachers.

4. Types of outcomes measured—knowledge, attitudes and practices.

### Dealing with missing data

If necessary, we will contact the corresponding authors of included studies to give us any missing data. We will describe missing data for each included study and discuss the extent to which the missing data could alter our results.

### Assessment of the risk of bias

The risk of bias for the included studies will be assessed using the modified Gates tool for observational studies [36] and Cochrane risk of bias tools for experimental studies [37]. We will use critical appraisal tool from NICE Public Health Guidance manual for 2013 [36] to assess the methodological quality of the qualitative studies included.

### Qualitative data analysis and synthesis

Qualitative synthesis for this review will be based on thematic synthesis of qualitative research. By examining the findings of each included study, key descriptive themes such as demographics, study design and findings of the studies on levels of knowledge, types of attitudes and practices on adolescent vaccination will be independently coded by two authors. Once all of the included studies has been examined and coded, the resulting themes and subthemes will be discussed within the study team to examine their relationship to the research questions. The qualitative synthesis will then proceed by using the ‘descriptive themes’ to develop ‘analytical themes’, which will be interpreted in reference to the research objective.

### Quantitative data analysis and synthesis

We will express the result of each study as a risk ratio with its corresponding 95% confidence intervals for dichotomous data or mean difference with its standard deviation for continuous data. We will group studies that compare broadly similar types of outcome to get feasible results on an overall estimate of effect. Log relative risks and standard errors of the log relative risk will be calculated for intervention studies. The log relative risks for intervention studies will be analysed together using the generic inverse variance method in Cochrane Review Manager. Random effects meta-analysis will be preferred due to anticipated heterogeneity in study results. If we encounter variation in reported outcome measures between studies, we will not pool the results but summarise the findings in a narrative format. Subgroup analyses may be conducted if possible, taking into account but not limited to age of target population, vaccine given, setting of the studies and country income status.

Both qualitative and quantitative findings will be interpreted taking into account the methodological quality of the studies and the strength of evidence. The basic principles of the GRADE approach will be applied to the synthesis of both quantitative and qualitative evidence [38].

### Subgroup analysis

Subgroup analyses may be conducted if possible, taking into account but not limited to age of target population, vaccine given, setting of the studies and country income status.

## Discussion

This review will include both quantitative and qualitative studies to identifying the evidence in the review. Although most systematic reviews rely on RCTs and other quantitative evidence, qualitative studies are being increasingly incorporated [39]. Some authors have expressed doubt about whether qualitative research can be systematically reviewed and synthesised due to often less specific outcome measures and more specific contextual detail [40]. However, qualitative research is valuable precisely because it reveals unique contextual detail and social processes, and consequently, data synthesis methods for qualitative reviews such as thematic analyses, meta-ethnography and realist reviews are being employed in the literature [41]. Qualitative methods are suited to an assessment of knowledge, beliefs and attitudes, and we hope that an attempt to synthesise such findings will add more nuance to understanding this issue in the African context.

This review will identify various levels of knowledge and attitudes as well as type of practices on adolescent vaccines among key role players in the study. This will enable us to understand how knowledge, attitudes and practices influence vaccine uptake and how it affects the vaccination coverage. We anticipate that our findings will be utilised to improve adolescent vaccination coverage in Africa through development of new policies on adolescent vaccination programmes.

### Strengths and limitations of this study

• To our knowledge, this is the first study that will attempt to use both quantitative and qualitative methods to assess and synthesise KAP on adolescent vaccination among parents, teachers and adolescents in Africa.

• The combination of qualitative and quantitative evidence in this study will make it more relevant and robust.

• A potential limitation of our study is high heterogeneity of studies and therefore not possible to conduct a meta-analysis.

### Definition of key terms

Knowledge refers to the understanding of any related topic on adolescent vaccines. Attitude refers to the feelings towards adolescent vaccines, as well as any preconceived ideas that one may have towards vaccination. Practice refers to the ways in which one demonstrates the knowledge and attitude (and any other influences) through actions.

## Competing interests

The authors declare that they have no competing interests.

## Authors’ contributions

All authors contributed to the conception and design of the protocol as follows. LHA, BMK, CSW and GDH conceived the study. LHA, BMK, TC and EA wrote the protocol with supervision from CSW and GDH. All authors read and approved the final manuscript.

## Supplementary Material

Additional file 1 **Appendices.** Appendix 1: Search strategy for PubMed database. Appendix 2: Data Extraction form: Knowledge, attitudes and practices on adolescent vaccination among parents, teachers and adolescents in Africa. Click here for file
