# Supplementary material for: Knowledge, attitudes and practices on adolescent vaccination among parents, teachers and adolescents in Africa: a systematic review protocol
Source: Syst Rev. 2014 Sep 9;3:100. doi: 10.1186/2046-4053-3-100 (PMC4161692; doi:10.1186/2046-4053-3-100)
Supplement: Additional file 1 — Appendices. Appendix 1: Search strategy for PubMed database. Appendix 2: Data Extraction form: Knowledge, attitudes and practices on adolescent vaccination among parents, teachers and adolescents in Africa. [file 2046-4053-3-100-S1.doc]

**Appendix 1**

**Search strategy for PubMed database.**

| Recent queries in PubMed (as of 20^th^ April 2014) | |
| --- | --- |
| Search | Query |
| #7 | ((((((((ALGERIA) OR (ANGOLA) OR (BENIN) OR (BOTSWANA) OR (BURKINA FASO) OR (BURUNDI) OR (CAMEROON) OR (CANARY ISLANDS OR "CANARY ISLANDS") OR ((CAPE VERDE) OR "CAPE VERDE") OR (CENTRAL AFRICAN REPUBLIC) OR (CHAD) OR (COMOROS) OR (CONGO) OR (DEMOCRATIC REPUBLIC CONGO) OR (DJIBOUTI) OR (EGYPT) OR ((EQUATORIAL GUINEA) OR "EQUATORIAL GUINEA") OR (ERITREA) OR (ETHIOPIA) OR (GABON) OR (GAMBIA) OR (GHANA) OR (GUINEA) OR ((GUINEA BISSAU) OR "GUINEA BISSAU") OR (IVORY COAST) OR ((COTE D'IVOIRE) OR "COTE D'IVOIRE") OR (KENYA) OR (LESOTHO) OR (LIBERIA) OR ((LIBYA) OR (LIBIA) OR (JAMAHIRIYA) OR (JAMAHIRYIA)) OR (MADAGASCAR) OR (MALAWI) OR (MALI) OR (MAURITANIA) OR (MAURITIUS) OR (MOROCCO) OR ((MOZAMBIQUE) OR (MOCAMBIQUE)) OR (NAMIBIA) OR (NIGER) OR (NIGERIA) OR (REUNION) OR (RWANDA) OR ((SAO TOME) OR "SAO TOME") OR (SENEGAL) OR (SEYCHELLES) OR ((SIERRA LEONE) OR "SIERRA LEONE") OR (SOMALIA) OR ((SOUTH AFRICA) OR "SOUTH AFRICA") OR ((ST HELENA) OR "ST HELENA") OR (SUDAN) OR (SWAZILAND) OR (TANZANIA) OR (TANGANYIKA) OR (TOGO) OR (TUNISIA) OR (UGANDA) OR ((WESTERN SAHARA) OR "WESTERN SAHARA") OR (ZAIRE) OR (ZAMBIA) OR (ZIMBABWE) OR (AFRICA[MH]) OR (SOUTH* AND AFRICA*) OR (WEST* AND AFRICA*) OR (EAST* AND AFRICA*) OR (NORTH* AND AFRICA*) OR (CENTRAL* AND AFRICA*) OR (SUB SAHARAN AFRICA*) OR (SUBSAHARAN AFRICA*) OR (AFRICA*) NOT (((GUINEA PIG*) OR "GUINEA PIG*") OR ((ASPERGILLUS NIGER) OR "ASPERGILLUS NIGERâ€))))) AND ((((("Vaccination"[Mesh]) OR vaccination) OR "Immunization"[Mesh]) OR immunization) OR immunisation)) AND ((("Adolescent"[Mesh]) OR adolescent) OR teenager)) AND ((accept) OR ((((((((((((((((Attitude to Health[MeSH Terms]) OR "Health Knowledge, Attitudes, Practice"[Mesh]) OR Health Knowledge, Attitudes, Practice) OR Patient Acceptance of Health Care[MeSH Terms]) OR acceptance) OR acceptability) OR knowledge) OR awareness) OR belief*) OR attitude) OR perception) OR adherence) OR compliance) OR willingness) OR uptake) OR understanding))) AND (((((((((((("MC-4 vaccine" [Supplementary Concept]) OR meningococcal-conjugate (MCV4)) OR meningococcal-conjugate) OR "Papillomavirus Vaccines"[Mesh]) OR "Influenza Vaccines"[Mesh]) OR "Tetanus"[Mesh]) OR tetanus) OR Influenza) OR ("Diphtheria"[Mesh] AND "Diphtheria-Tetanus-acellular Pertussis Vaccines"[Mesh])) OR "Pertussis Vaccine"[Mesh]) OR Diphtheria-Tetanus-acellular Pertussis Vaccines) OR "Influenza, Human"[Mesh]) Sort by: [relevance] |
| #6 | (((ALGERIA) OR (ANGOLA) OR (BENIN) OR (BOTSWANA) OR (BURKINA FASO) OR (BURUNDI) OR (CAMEROON) OR (CANARY ISLANDS OR "CANARY ISLANDS") OR ((CAPE VERDE) OR "CAPE VERDE") OR (CENTRAL AFRICAN REPUBLIC) OR (CHAD) OR (COMOROS) OR (CONGO) OR (DEMOCRATIC REPUBLIC CONGO) OR (DJIBOUTI) OR (EGYPT) OR ((EQUATORIAL GUINEA) OR "EQUATORIAL GUINEA") OR (ERITREA) OR (ETHIOPIA) OR (GABON) OR (GAMBIA) OR (GHANA) OR (GUINEA) OR ((GUINEA BISSAU) OR "GUINEA BISSAU") OR (IVORY COAST) OR ((COTE D'IVOIRE) OR "COTE D'IVOIRE") OR (KENYA) OR (LESOTHO) OR (LIBERIA) OR ((LIBYA) OR (LIBIA) OR (JAMAHIRIYA) OR (JAMAHIRYIA)) OR (MADAGASCAR) OR (MALAWI) OR (MALI) OR (MAURITANIA) OR (MAURITIUS) OR (MOROCCO) OR ((MOZAMBIQUE) OR (MOCAMBIQUE)) OR (NAMIBIA) OR (NIGER) OR (NIGERIA) OR (REUNION) OR (RWANDA) OR ((SAO TOME) OR "SAO TOME") OR (SENEGAL) OR (SEYCHELLES) OR ((SIERRA LEONE) OR "SIERRA LEONE") OR (SOMALIA) OR ((SOUTH AFRICA) OR "SOUTH AFRICA") OR ((ST HELENA) OR "ST HELENA") OR (SUDAN) OR (SWAZILAND) OR (TANZANIA) OR (TANGANYIKA) OR (TOGO) OR (TUNISIA) OR (UGANDA) OR ((WESTERN SAHARA) OR "WESTERN SAHARA") OR (ZAIRE) OR (ZAMBIA) OR (ZIMBABWE) OR (AFRICA[MH]) OR (SOUTH* AND AFRICA*) OR (WEST* AND AFRICA*) OR (EAST* AND AFRICA*) OR (NORTH* AND AFRICA*) OR (CENTRAL* AND AFRICA*) OR (SUB SAHARAN AFRICA*) OR (SUBSAHARAN AFRICA*) OR (AFRICA*) NOT (((GUINEA PIG*) OR "GUINEA PIG*") OR ((ASPERGILLUS NIGER) OR "ASPERGILLUS NIGERâ€))) |
| #5 | ((((((((((("MC-4 vaccine" [Supplementary Concept]) OR meningococcal-conjugate (MCV4)) OR meningococcal-conjugate) OR "Papillomavirus Vaccines"[Mesh]) OR "Influenza Vaccines"[Mesh]) OR "Tetanus"[Mesh]) OR tetanus) OR Influenza) OR ("Diphtheria"[Mesh] AND "Diphtheria-Tetanus-acellular Pertussis Vaccines"[Mesh])) OR "Pertussis Vaccine"[Mesh]) OR Diphtheria-Tetanus-acellular Pertussis Vaccines) OR "Influenza, Human"[Mesh] |
| #4 | (accept) OR ((((((((((((((((Attitude to Health[MeSH Terms]) OR "Health Knowledge, Attitudes, Practice"[Mesh]) OR Health Knowledge, Attitudes, Practice) OR Patient Acceptance of Health Care[MeSH Terms]) OR acceptance) OR acceptability) OR knowledge) OR awareness) OR belief*) OR attitude) OR perception) OR adherence) OR compliance) OR willingness) OR uptake) OR understanding) |
| #3 | (((((((((((((((Attitude to Health[MeSH Terms]) OR "Health Knowledge, Attitudes, Practice"[Mesh]) OR Health Knowledge, Attitudes, Practice) OR Patient Acceptance of Health Care[MeSH Terms]) OR acceptance) OR acceptability) OR knowledge) OR awareness) OR belief*) OR attitude) OR perception) OR adherence) OR compliance) OR willingness) OR uptake) OR understanding |
| #2 | (("Adolescent"[Mesh]) OR adolescent) OR teenager |
| #1 | (((("Vaccination"[Mesh]) OR vaccination) OR "Immunization"[Mesh]) OR immunization) OR immunisation |

**Web sites and databases for grey materials:**

World Health Organisation (WHO) (http://www.who.int/), Global Alliance for Vaccine and Immunization (GAVI) (http://www.gavialliance.org/), United Nation Children’s Funds (UNICEF) (http://www.unicef.org/), PATH Vaccine Resources Library (http://www.path.org/), US Centers for Disease Control and Prevention (CDC) (http://www.cdc.gov/), The communication initiative network (http://www.comminit.com/), and Immunization basics (http://www.immunizationbasics.jsi.com/Index.html)

Appendix 2: Data Extraction form: Knowledge, attitudes and practices on adolescent vaccination among parents, teachers and adolescents in Africa.

| **Study ID** *(surname of first author and year first full report of study was published e.g. Smith 2001)* |
| --- |
|  |

1. General Information

| **Date form completed** *(dd/mm/yyyy)* |  |
| --- | --- |
| **Name/ID of person extracting data** |  |
| **Reference citation** |  |
| **Study author contact details** |  |
| **Publication type**  *(e.g. full report, abstract, letter)* |  |
| **References of potentially eligible studies from the reference list** |  |
| **Notes:** | |

1. Study characteristics

| **Study Characteristics** | **Eligibility criteria**  *(Insert inclusion criteria for each characteristic as defined in the Protocol)* | | **Location in text or source** *(pg & ¶/fig/table/other)* | | |  |
| --- | --- | --- | --- | --- | --- | --- |
|  |  |  |
| **Vaccine and disease targeted** |  | |  | | | |
| **Location** |  | |  | | | |
| **Study design** |  | |  | | | |
| **Participants** |  | |  | | | |
| **Types of outcome measures** |  | |  | | | |
| **INCLUDE** ☐ | | **EXCLUDE** ☐ | | | | |
| **Reason for exclusion** |  | | | | | |
| **Notes:** | | | | | | |

**DO NOT PROCEED IF STUDY EXCLUDED FROM REVIEW**

1. **Characteristics of included studies**

Methods

|  | **Descriptions as stated in report/paper** | | **Location in text or source** *(pg & ¶/fig/table/other)* |
| --- | --- | --- | --- |
| **Aim of study** |  | |  |
| **Study Design** |  | |  |
| **Unit of analysis**  *(by individuals / groups or focus)* |  | |  |
| **Start date** |  | |  |
| **End date** |  | |  |
| **Duration of participation**  *(from recruitment to last follow-up)* |  | |  |
| **Study characteristics** | ☐ Qualitative study characteristics (If yes, proceed to section E)  ☐ Quantitative study characteristics (If yes, proceed to section D) | |  |
| **Ethical approval needed/ obtained for study** | **☐ ☐ ☐**  Yes No Unclear |  |  |
| **Notes:** | | | |

1. **Quantitative study characteristics**

**Populati**on and settings

|  | **Description**  *Include comparative information for each intervention or comparison group if available* | | **Location in text or source** *(pg & ¶/fig/table/other)* |
| --- | --- | --- | --- |
| **Population description**  *(from which study participants are drawn)* |  | |  |
| **Setting**  *(including location and social context)* |  | |  |
| **Inclusion criteria** |  | |  |
| **Exclusion criteria** |  | |  |
| **Method of recruitment of participants** *(e.g. phone, mail, clinic patients)* |  | |  |
| **Informed consent obtained** | **☐ ☐ ☐**  Yes No Unclear |  |  |
| **Notes** |  | | |

Participants

|  | **Description as stated in paper** | **Location in text** |
| --- | --- | --- |
| **Total no. enrolled** |  |  |
| **Withdrawals and exclusions** |  |  |
| **Age** |  |  |
| **Sex** |  |  |
| **Notes** |  | |

**Outcome measures**

**Vaccination uptake**

|  | **Description as stated in report/paper** | **Location in text or source** *(pg & ¶/fig/table/other)* |
| --- | --- | --- |
| **Vaccine** |  |  |
| **Disease** |  |  |
| **country** |  |  |
| **Routine immunization program for adolescents with specific vaccine** | **☐** Yes **☐** No  If Yes specify |  |
| **Other vaccine program in place for adolescents** | **☐** Yes **☐** No  If Yes specify |  |
| **Vaccination coverage among adolescents** |  |  |
| **Prevalence of vaccine disease preventable diseases among adolescents or adults** |  |  |
| **Incidence of vaccine preventable diseases among adolescents or adults** |  |  |
| **Knowledge on adolescent vaccine** |  |  |
| **Attitudes on adolescent vaccine** |  |  |
| **Practices on adolescent vaccine** |  |  |
| **Notes:** | | |

Risk of bias assessment

| **Does the study have a high risk of:** | **Description and assessment** |
| --- | --- |
| **Selection bias?**  **(allocation concealment , allocation sequence** |  |
|  | **☐Yes ☐No ☐Unclear** |
| **Attrition bias?** |  |
|  | **☐Yes ☐No ☐Unclear** |
| **Reporting bias?** |  |
|  | **☐Yes ☐No ☐Unclear** |
| **Are study results valid?** |  |
|  | **☐Yes ☐No ☐Unclear** |

Other relevant information

|  | **Descriptions/figures as stated in report/paper/book chapter** | **Reference page/table or figure in the study** |
| --- | --- | --- |
| **Key conclusions from the authors** |  |  |
| **Notes** |  |  |

1. **Qualitative study characteristics**

|  | **Description as stated in report/paper** | **Location in text or source** *(pg & ¶/fig/table/other)* |
| --- | --- | --- |
| **Overall aim/purpose** |  |  |
| **Research/analytical question(s)** |  |  |
| **Methodology** |  |  |
| **Geographical setting** |  |  |
| **Cultural setting/social context** |  |  |
| **Participants** |  |  |
| **Data collection method: Survey, Interviews, Focus group discussions.** |  |  |
| **Outcome** |  |  |
| **Thematic analysis** |  |  |
| **Overall perception of adolescent vaccine and disease** |  |  |
| **Notes:** | | |

**Chec**klist for qualitative studies

| **Clear statement or aims of the research:** | | | |
| --- | --- | --- | --- |
|  | **Description and assessment** | **Comments** | **Location in text or source** *(pg & ¶/fig/table/other)* |
| **Type of qualitative study** | **☐** Participant observation  ☐ Open- ended interviews  ☐Structured interviews  **☐** Others (specify) |  |  |
| **Theoretical approach**  **1. Is a qualitative approach appropriate?**  For example:   - Does the research question seek to understand processes or structures, or illuminate subjective experiences or meanings?   Could a quantitative approach better have addressed the research question? | **☐** Appropriate  **☐** Inappropriate  **☐** Not sure |  |  |
| **2. Is the study clear in what it seeks to do?**  For example:   - Is the purpose of the study discussed – aims/objectives/research question/s? - Is there adequate/appropriate reference to the literature? - Are underpinning values/assumptions/theory discussed? | ☐Clear  **☐** Unclear  **☐** Mixed |  |  |
| **Study design**  **3. How defensible/rigorous is the research design/methodology?**  For example:   - Is the design appropriate to the research question? - Is a rationale given for using a qualitative approach? - Are there clear accounts of the rationale/justification for the sampling, data collection and data analysis techniques used? - Is the selection of cases/sampling strategy theoretically justified? | ☐ Defensible  ☐ Indefensible  ☐ Not sure |  |  |
| **Data collection**  **4. How well was the data collection carried out?**  For example:   - Are the data collection methods clearly described? - Were the appropriate data collected to address the research question? - Was the data collection and record keeping systematic? | ☐ Appropriately  ☐ Inappropriately  ☐ Not sure/inadequately reported |  |  |
| **Trustworthiness**  **5. Is the role of the researcher clearly described?**  For example:   - Has the relationship between the researcher and the participants been adequately considered? - Does the paper describe how the research was explained and presented to the participants? | **☐** Clearly described  **☐** Unclear  **☐** Not described |  |  |
| **6. Is the context clearly described?**  For example:   - Are the characteristics of the participants and settings clearly defined? - Were observations made in a sufficient variety of circumstances - Was context bias considered | **☐** Clear  **☐** Unclear  ☐ Not sure |  |  |
| **7. Were the methods reliable?**  For example:   - Was data collected by more than one method? - Is there justification for triangulation, or for not triangulating? - Do the methods investigate what they claim to? | **☐** Reliable  **☐** Unreliable  **☐** Not sure |  |  |
| **Analysis**  **8. Is the data analysis sufficiently rigorous?**  For example:   - Is the procedure explicit – i.e. is it clear how the data was analysed to arrive at the results? - How systematic is the analysis, is the procedure reliable/dependable?   Is it clear how the themes and concepts were derived from the data? | ☐ Rigorous  ☐ Not rigorous  ☐ Not sure/not reported |  |  |
| **9. Is the data 'rich'?**  For example:   - How well are the contexts of the data described? - Has the diversity of perspective and content been explored? - How well has the detail and depth been demonstrated? - Are responses compared and contrasted across groups/sites? | **☐** Rich  **☐** Poor  ☐Not sure/not reported |  |  |
| **10. Is the analysis reliable?**  For example:   - Did more than 1 researcher theme and code transcripts/data? - If so, how were differences resolved? - Did participants feedback on the transcripts/data if possible and relevant? - Were negative/discrepant results addressed or ignored? | **☐** Reliable  **☐** Unreliable  **☐** Not sure/not reported |  |  |
| **11. Are the findings convincing?**  For example:   - Are the findings clearly presented? - Are the findings internally coherent? - Are extracts from the original data included? - Are the data appropriately referenced? - Is the reporting clear and coherent? | **☐** Convincing  **☐** Not convincing  **☐** Not sure |  |  |
| **12. Are the findings relevant to the aims of the study?** | ☐ Relevant  **☐** Irrelevant  **☐** Partially relevant |  |  |
| **13. Conclusions**  For example:   - How clear are the links between data, interpretation and conclusions? - Are the conclusions plausible and coherent? - Have alternative explanations been explored and discounted? - Does this enhance understanding of the research topic? - Are the implications of the research clearly defined?   **Is there adequate discussion of any limitations encountered?** | **☐** Adequate  **☐** Inadequate  **☐** Not sure |  |  |
| **14. How clear and coherent is the reporting of ethics?**  For example:   - Have ethical issues been taken into consideration? - Are they adequately discussed e.g. do they address consent and anonymity? - Have the consequences of the research been considered i.e. raising expectations, changing behaviour? - Was the study approved by an ethics committee? | **☐** Appropriate  **☐** Inappropriate  **☐** Not sure/not reported |  |  |

Other relevant information

|  | **Descriptions/figures as stated in report/paper/book chapter** | **Reference page/table or figure in the study** |
| --- | --- | --- |
| **Key conclusions from the authors** |  |  |
| **Notes** |  |  |
